# Stretching Before Resistance Training as a Strategy to Improve Stair Descent Performance in Older Women

**DOI:** 10.3390/sports13080276

**Published:** 2025-08-20

**Authors:** Vittoria Ferrando, Marco Panascì, Ambra Bisio, Valentina Chiarotti, Federica Marmondi, Matteo Bonato, Piero Ruggeri, Emanuela Faelli

**Affiliations:** 1 Department of Experimental Medicine, Section of Human Physiology, University of Genoa, 16132 Genoa, Italy; vittoria.ferrando@unige.it (V.F.); ambra.bisio@unige.it (A.B.); piero.ruggeri@unige.it (P.R.); emanuela.faelli@unige.it (E.F.); 2 Centro Polifunzionale di Scienze Motorie, University of Genoa, 16126 Genoa, Italy; valentina.chiarotti.iic98@gmail.com (V.C.); federica.marmondi@edu.unige.it (F.M.); 3 Department of Neuroscience, Rehabilitation, Ophthalmology, Genetics and Maternal Child Health, University of Genoa, 16126 Genoa, Italy; 4 Department of Biomedical Sciences for Health, Università degli Studi di Milano, 20122 Milan, Italy; matteo.bonato@unimi.it; 5 Laboratory of Movement and Sport Sciences (LaMSS), IRCCS Istituto Ortopedico Galeazzi, 20161 Milan, Italy

**Keywords:** older adults, warm-up, stretching, resistance training, flexibility, stair decent performance

## Abstract

**Background**: Aging is associated with reduced joint flexibility and balance, which increases the risk of falls, especially during stair descent where motor control is critical. Stretching has been shown to improve ankle range of motion and gait speed. This study investigated the effects of a 4-week training program combining stretching plus resistance training (RT) with elastic bands on functional capacity and ankle stability during stair descent in older women. **Methods**: Twenty-four active older women (mean age: 73.1 ± 0.97 years) were randomly assigned to static stretching (SS), dynamic stretching (DS) and control (CG) groups. All participants completed two weekly 60 min sessions consisting of progressive RT preceded by three different warm-ups. The SS and DS groups completed static or dynamic stretching, while the CG walked. Assessments included 30s-Chair Stand (30s-CS), Handgrip Strength (HGS), Time Up and Go (TUG), Chair Sit and Reach (CSR), Rating of Perceived Exertion (RPE), and ankle kinematics during stair descent. **Results**: All groups improved 30s-CS and TUG (*p* < 0.05). Only the SS group improved CSR in both legs and the ankle dorsiflexion angle during stair descent at final foot contact (*p* = 0.002). RPE increased over time across all groups (*p* < 0.0001); however, the SS and DS groups reported lower exertion than the CG group in first–second weeks (*p* = 0.0001–0.003). **Conclusions**: SS prior to progressive RT improved flexibility and ankle kinematics during stair descent, thus reducing the perception of effort particularly during the initial training phase. These findings indicate the effectiveness of SS as a warm-up strategy for increasing ROM and potentially reducing the risk of falls in this population.

## 1. Introduction

Aging is a natural and biological process that leads to progressive decline in muscle strength, joint mobility and postural control, particularly in the lower limbs. These impairments have been shown to reduce functional capacity, thus compromising independence in older adults [1,2]. While aging itself is the main cause for the decline, additional factors such as a sedentary lifestyle can accelerate the degenerative process.

Moreover, muscle mass decreases with advancing age due to a reduction in fiber, muscle size and number, as well as a shift in muscle fiber type composition, particularly a decrease in type II fibers, and reduced neural activation. All of these factors impair movement efficiency [3].

One notable consequence is a reduction in joint range of motion (ROM), which decreases by approximately six degrees per decade after the age of 55 [4]. This decline negatively affects balance and increases the risk of falls and related injuries [1,4,5,6]. This risk is particularly pronounced during stair descent, which is a demanding motor task requiring precise eccentric control of the ankle joint to regulate body mass transfer between steps. Older adults often perform this task near their maximum eccentric capacity, which limits their ability to respond to unexpected perturbations [7,8,9]. Bosse et al. [10] observed that older adults perform stair descent closer to their maximal eccentric capacity compared to younger individuals, suggesting a diminished ability to generate sufficient joint movements necessary for the safe control of the center of mass during descent. To compensate, older adults typically adopt conservative strategies such as reducing vertical swing and increasing downward limb velocity [7]; however, these compensations are often inadequate due to limited eccentric ankle force generation [8]. Furthermore, the overall synergy of the lower limb encompassing hip, knee, and ankle coordination plays a critical role in force application and movement stability. Previous biomechanical evidence investigated stair descent performance in older adults, using different methods to evaluate this aspect.

For instance, Hamel et al. (2004) [11] reported that older adults showed an exaggerated frontal plane movement of the upper body during stair descent when compared to ascent, while Lee et al. (2006) [12] found that elderly adults showed a significantly greater medial inclination angle during the stair-to-floor transition phase when compared to young adults, thus compromising their ability to regulate body sway during this task. Moreover, a recent study by Kováčiková and colleagues (2021) [13] which examined the gender and age-related differences in balance control during and after stair descent on a foam mat, suggest that with advancing age, men are at higher risk of forward falls, whereas women are at higher risk of forward and sideways falls.

However, in stair descent balance control, physical activity could play a significant role.

In this context, Resistance training (RT) has been proven to effectively counteract age-related decline by enhancing muscle strength, postural stability, and overall functionality, particularly in motor tasks such as descending stairs [14,15]. Progressive RT, which involves gradually increasing the resistance load as strength improves, has been especially beneficial. Studies indicate that two to three sessions per week can significantly improve muscle function, balance, gait speed, and functional performance in older people [16,17]. Among RT modalities, elastic band training offers a safe, accessible, and cost-effective alternative to traditional weight-based protocols, producing comparable results in terms of improving strength [18], flexibility [19,20], and quality of life, promoting functional independence in older people [8,18,21], and reducing the risk of injury [18,22].

Flexibility is another crucial component of physical fitness in older adults. Among the different types of stretching, static stretching (SS) is one of the most commonly employed modalities to improve flexibility [23], improving joint ROM [24] and in reducing muscle-tendon stiffness, especially in the hamstrings [25] in older adults. Acute gains are mediated by Golgi tendon organs [26], while chronic improvements are related to reduced stiffness and neural adaptations [27]. These improvements are particularly relevant for tasks that require controlled lowering of the body [4], such as descending stairs [6,8]. During this task, the muscle-tendon units of the lower limbs are involved in eccentric contractions to absorb the mechanical load [7,8], with the ankle joint playing a crucial role in maintaining stability, balance, and reducing the potential risk of falls [8,28,29]. Dynamic stretching (DS), characterized by active and controlled movements that progressively take joints through their full ROM, has gained interest as an effective strategy to enhance mobility and functional readiness in older adults [30]. Unlike SS, DS may better prepare the neuromuscular system for functional movements by enhancing proprioception, raising core temperature and promoting muscle activation [31]. Studies suggest that DS can improve balance, mobility, and muscular coordination in older individuals, potentially offering added benefits for dynamic stability [30,32].

Despite the known risks associated with stair descent, few studies have examined how pre-resistance stretching may influence ankle control and perceived exertion during this task.

In this context, a combination of resistance and stretching exercises may be particularly effective in improving postural stability, and enhancing joint mobility, thus increasing functional independence in older adults. Given these considerations, the present study investigated the chronic effects of a combined 4-week program combining progressive RT and static or dynamic stretching exercises on ankle stability, functional performance and perceived exertion during stair descent, in active older women.

We hypothesized that including stretching within the warm-up, followed by progressive RT, would positively influence functional capacity and ankle stability during stair descent in older women. In addition, given the benefits of stretching exercises on muscle function, it is reasonable to hypothesize that they could effectively decrease the perception of effort after training sessions.

## 2. Materials and Methods

### 2.1. Participants

An a priori sample size estimation was based on a 30 s Chair Stand (30s-CS) as the primary outcome, as it is considered a reliable and valid indicator of lower body strength in active older adults. Sample size was estimated using the G*Power software (3.1 software Düsseldorf, Germany) applying repeated measures ANOVA (F Test) with α = 0.05, power of 80% and an effect size (ES) of 0.70, allowing for a 15% margin. The estimated sample size was at least 21 participants.

Twenty-four active older women were enrolled in the study. Inclusion criteria were as follows: women aged ≥65 years, a valid medical certificate for physical activity, and no recent musculoskeletal and neuromuscular injuries. Participants were considered physically active in accordance with the World Health Organization guidelines, which define older adults as physically active when they engage in at least 150 min of moderate-intensity physical activity per week [33].

Moreover, to exclude sarcopenic individuals, participants were screened using bioelectrical impedance analysis (BIA) and handgrip strength (HGS). The skeletal muscle index (SMI), calculated using Janssen at al.’s [34] formula, had to be <6.75 kg/m^2^, and HGS > 20 kg [35] (See Preliminary Assessments section).

After their initial enrolment, participants were randomized and allocated with a 1:1:1 ratio to one of three groups: static stretching plus RT (SS group), dynamic stretching plus RT (DS group), or control group performing RT alone (CG group). A computer-generated blocked randomization list of random numbers was used for participant allocation.

The experimental protocol was approved by the Ethics Committee of University of Milan (protocol code: 110/24 and date of approval: 14 October 2024) conforming to the code of Ethics of the World Medical Association (Declaration of Helsinki). Written informed consent was obtained from all study participants.

### 2.2. Study Design

The 4-week open-label study involved randomly dividing the enrolled older women into two experimental groups (static stretching, SS; dynamic stretching, DS) and one control group (CG). The warm-up routine differed between these three groups.

In the SS group, stretching exercises were performed until reaching the point of discomfort (POD), defined as the sensation of mild to moderate tension without pain; whereas in the DS group, exercises were aimed at achieving the maximum available range of motion (ROM) for each targeted muscle group, emphasizing controlled and active movements throughout the stretch. In contrast, in the CG group, participants executed a brisk walk.

The study began with preliminary assessments, in which the sarcopenic state was analyzed, the training protocol was explained, and the informed consent form was completed and signed. Before (PRE) and at the end of the intervention period (POST), subjects underwent functional testing and analysis of ankle movement during stair descent.

The choice to use different tests was influenced by their ability to assess different motor skills and aspects of functional fitness such as strength, mobility, and flexibility. Moreover, the administration sequence was designed to minimize fatigue and ensure participant safety, starting with the most physically demanding (strength), followed by mobility, and ending with the least demanding (flexibility), which also served as a cooldown.

**Figure 1:**
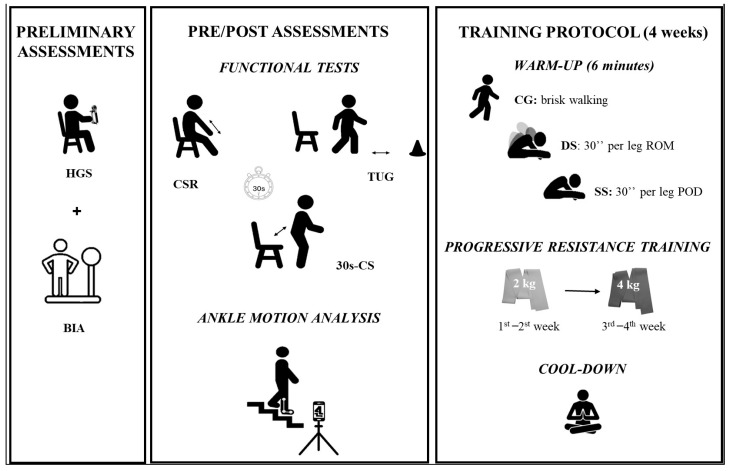
Participants underwent preliminary assessments, after which, before (PRE) and after (POST) the training protocol, all groups underwent functional tests and ankle kinematics during stair descent. All groups performed the same 4-week training protocol, consisting of a warm-up, progressive resistance training with elastic bands, and cool down. HGS: Handgrip Strength; BIA: Bioimpedance Analysis; CSR: Chair Sit and Reach; TUG: Time Up and Go; 30s-CS: 30 s Chair Stand; CG: Control Group; DS: Dynamic Stretching Group; SS: Static Stretching Group; ROM: Range of Movement; POD: Point of Discomfort.

All training sessions and tests were conducted under the supervision of a sports science researcher. The experimental protocol is illustrated in Figure 1.

### 2.3. Training Protocol

During the training protocol, all groups (SS, DS and CG) performed 8 training sessions, twice a week, for a total duration of approximately 60 min. Each session consisted of a 6 min warm-up, 45 min of progressive resistance training, and a 5 min cool down.

#### 2.3.1. Warm-Up

The 6 min warm-up routine differed between the experimental groups, with each group having its own unique routine. The SS and DS groups performed stretching exercises while the CG group executed a brisk walk.

Both the SS and DS groups performed 6 min of stretching exercises designed to stretch the hamstrings, hip adductors, gluteus, quadriceps, calves, and soleus [4,36,37]. Both SS and DS exercises focused on the same muscle groups and followed the same sequence. In particular, to stretch the hamstrings, participants sat on the floor with one leg extended and the other flexed, placing the foot placed against the knee of the extended leg and reaching for the toe of the extended leg. To stretch the hip adductors, participants sat on the floor with the soles of their feet touching each other, their hips in abduction and their knees flexed; using one hand for support, they gently pushed one knee towards the floor. To stretch their gluteus, participants lay on their backs, flexed their knee and hip joints to bring the thigh closer to the abdominal region and kept the opposite leg extended and in contact with the floor. The quadriceps stretch was performed in a standing position by bending the knee and grasping the ankle with the corresponding hand, pulling it gently towards the gluteus until they felt a stretching sensation in the front part of the thigh. The calf stretch was performed by participants from a standing position with their hands on the wall, one leg extended and the other positioned with the toe on the back edge of a 30 cm platform that was in contact with the wall. To stretch the soleus, participants had to stand with their hands resting on a wall for support, bend both knees and keep the heel of the back foot firmly on the ground, while tilting their trunk slightly forward. Each SS exercise lasted 30 s per leg and was performed to the ‘point of discomfort’ (100% POD) without inducing pain; whereas DS exercises involved dynamic movements. Specifically, each DS exercise was repeated 30 s per leg, with the aim of achieving a maximum range of motion (ROM) with each repetition and returning to the starting position [36]. DS exercises were performed at a controlled velocity, with the assistance of a digital metronome set to 30 beats.min^−1^ (0.5 Hz) [36].

All stretching exercises were carried out by a sport science researcher to ensure proper technique, timing, and safety, particularly for DS exercises which require controlled movement. Supervision was especially important given the functional limitations of the older adult population.

#### 2.3.2. Progressive Resistance Training

**Figure 2:**
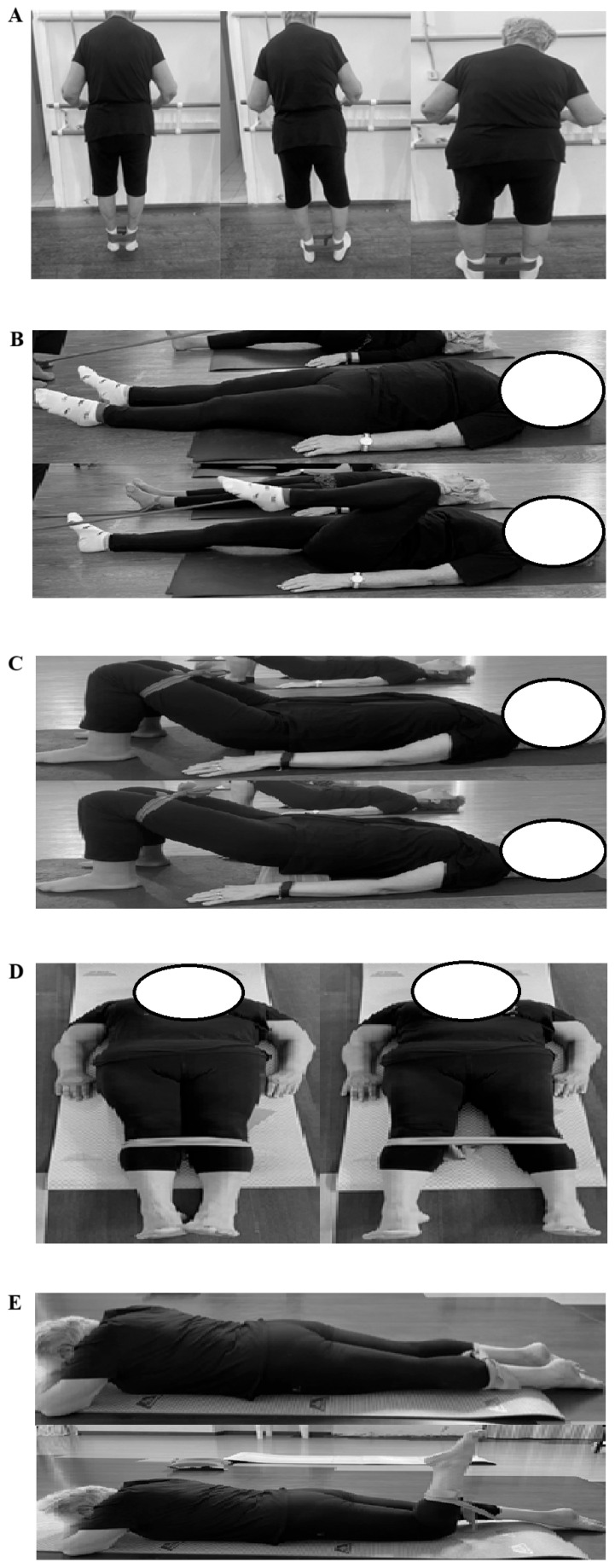
Resistance exercises with elastic bands. (**A**) Standing calf with hip abduction; (**B**) Supine hip flexion with elastic band; (**C**) Gluteal bridge; (**D**) Hip abduction with elastic from supine; (**E**) Hamstring curl from a prone position.

All groups performed the same lower limb progressive RT. Specifically, resistance exercises were performed with elastic bands and included (i) standing calf with hip abduction, (ii) supine hip flexion with elastic band, (iii) gluteal bridge, (iv) hip abduction with elastic from supine, (v) hamstring curl from a prone position [5]. Progressive resistance exercises are presented in Figure 2. The intensity of the training gradually increased after the first two weeks, starting with elastic bands that initially had a load of 2 kg, and then moving on to elastic bands that had a load of 4 kg. Each exercise was performed under the supervision of a certified specialist to ensure both safety and adherence. The protocol required that each exercise be carried out in 3 sets of 10 repetitions, with a one-minute rest period between each set. Each repetition lasted 4 s, divided into 2 s for the eccentric phase and 2 s for the concentric phase [38]. A repetition tempo of 2 s per eccentric phase and 2 s per concentric phase was adopted to ensure controlled execution and enhance time under tension. This choice is supported by evidence showing that older adults have greater eccentric strength, which provides a functional reserve, and that exercises focusing on the eccentric phase impose lower stress, making them both safe and effective for this population [39].

At the end of each session, participants reported their perceived exertion using the Borg CR-10 Rating of Perceived Exertion (RPE) scale [18,40]. Before the intervention began, all participants were familiarized with the exercises and the Borg scale.

#### 2.3.3. Cool-Down

At the end of each training session, all participants performed the same cool-down consisting of 5 min of breathing, knee extension, and ankle movement exercises [41].

### 2.4. Measures

#### 2.4.1. Preliminary Assessments

Body composition parameters required for the application of the Janssen formula were measured using bioelectrical impedance (BIA; Tanita, BC-420 MA, Tanita, Tokyo, Japan) at a frequency of 50 kHz. The measurements were performed at least 2 h after the meal to ensure optimal accuracy. Additionally, the general strength of each participant was measured using the HG dynamometer (Sammons Preston, Rolyan, Bolingbrook, IL, USA), which provides a standardized and objective assessment of arm strength. To obtain accurate and reliable measurements, three tests were performed on each side and the values from both were then averaged to obtain the total HGS test [35].

#### 2.4.2. Functional Tests

*30 s Chair Stand test.* To test the explosive strength of the lower limbs of the participants, in accordance with a previous study [42], the 30 s chair stand test (30s-CS) was utilized. This test was administered using an armless chair positioned against a wall to prevent it from moving. Participants started seated in a standard chair (seat height of 40 cm), with their back upright, their feet on a flat surface positioned about shoulder width apart, their arms crossed at the wrists and held against the chest, and their hips and knees flexed at approximately 90° [17]. Then, they were instructed to stand up and sit down as many times as possible within 30 s. The total number of full stands completed within the time limit was recorded as the final score [42].

*Time Up and Go test.* To assess static and dynamic balance, the risk of falling and gait speed, the Time Up and Go test (TUG) was used. For the execution, a chair, a stopwatch, and a cone to mark 3 m distance from the chair are required. The subject begins in a seated position, then stands up on the examiner’s command. They must walk for 3 m, turn 180° around the cone, return to the chair, and sit down. The time stopped when the patient was completely seated [43].

*Chair Sit and Reach test.* Posterior kinetic chain flexibility was assessed using the Chair Sit and Reach test (CSR). The test requires a chair and a tape measure. Each subject sits on the edge of the chair with one foot on the floor and the other leg extended with the heel on the floor and the ankle flexed at 90°; the subject places one hand on the top of the other with the middle fingers overlapping and bends forward at the hips to reach for their toes. The examiner measures the distance between the tip of the middle fingers and the tip of the toes using a tape measure. The test was performed on both the right and left leg [44].

#### 2.4.3. Ankle Motion Analysis

To evaluate the effectiveness of stretching protocols incorporated into the warm-up on dorsiflexion range of motion, a video analysis of stair descent was conducted before (PRE) and after (POST) the training intervention. All participants performed the stair descent task using the same stair configuration and under consistent lighting conditions. Marker placement was conducted and verified by the same sport science researcher for all trials to ensure methodological consistency and accuracy of motion capture data.

**Figure 3:**
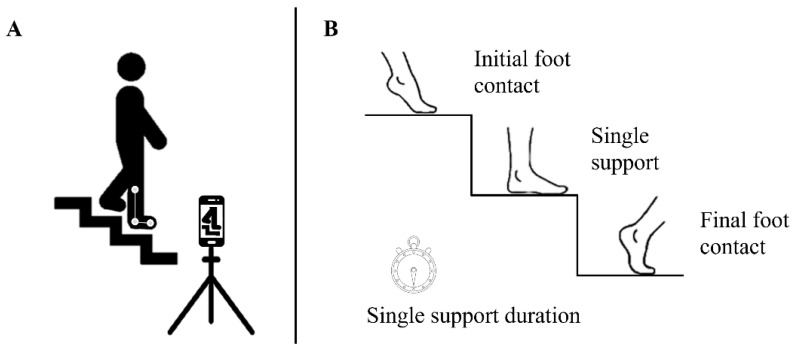
Ankle motion analysis (**A**) cameras setting position for stair descent; (**B**) ankle parameters: initial foot contact, single support, final foot contact, and single support duration.

Specifically, ankle joint kinematics were videorecorded from a lateral perspective as participants descended three steps of a staircase. Participants were instructed to wear black pants to facilitate the identification and marking of landmarks on the right lower limb. Three spherical markers were positioned on the lateral femoral epicondyle, the lateral fibular malleolus, and the center of the fifth metatarsophalangeal joint [6]. Each descent was recorded using an iPhone 11 Pro (Apple Inc., Cupertino, CA, USA) at 60 frames per second (fps) with 4K resolution. The device was placed laterally at a distance of 1.3 m from the women, ensuring the ankle joint remained within the frame. The stair used for the descent trials consisted of steps having a tread depth of 285 mm, a width of 900 mm, and a standard rise of 170 mm. Participants took turns positioning themselves at the fourth step from the floor and they were instructed to start their descent with the left leg ensuring that the right foot would make contact with the second step, designated as the reference step for analysis (Figure 3A). The captured trials were subsequently analyzed using Kinovea software 0.8.15 (Copyright © 2006–2011, Joan Charmant & Contrib, France). Three key frames were selected from each video to assess the ankle joint angles: *initial foot contact*—when the right foot first touched the second step, *single support*—when the subject was supported entirely by the right leg with the left foot lifted off the first step, and *final foot contact*—the moment just before the right foot was completely disengaged from the step. In addition, a stopwatch was used to measure the *single support duration*—the time elapsed between the moment the subject started to descend with their left foot and the moment they made first contact with the step (Figure 3B).

#### 2.4.4. Rating Perceived Exertion (RPE)

Perceived personal exertion was measured using the CR-10 version of the Borg Rating of Perceived Exertion (RPE) scale [40]. Prior to the data collection, all participants were thoroughly familiarized with the scale and its anchoring procedures, to ensure consistent and accurate reporting. RPE scores were recorded at two standardized time points during each experimental session: two minutes after warm-up (POSTwarm-up) [36], and two minutes after completion of resistance exercises (POSTexercise) [45]. At each of these time points, participants rated their perceived exertion by selecting a value from 0 to 10 on the CR-10 scale, corresponding to the intensity of the effort they experienced [36]. The weekly RPE values were obtained by averaging the values measured during each week’s two training sessions.

### 2.5. Statistical Analysis

All data were analyzed using linear mixed model analysis [46], with GROUP specified as a fixed effect and TIME as a random effect, reflecting the study’s structure (Level 1: individuals; Level 2: time). When significant interaction was observed, Bonferroni post hoc tests were conducted. Effect sizes were reported using eta squared (ƞ^2^). Data are presented as means ± standard error (SE), and 95% confidence intervals (CI) are provided when appropriate. Statistical significance was set at *p* = 0.05. All analyses were conducted using IBM SPSS Statistic, version 20 for Windows.

## 3. Results

### 3.1. Study Population

Twenty-four active older women (mean age: 73.1 ± 0.97 years), all with several years of participation in adapted physical activity (APA) were recruited. Baseline characteristics are shown in Table 1. Randomization ensured baseline comparability among groups, with no significant differences between groups.

The baseline characteristics table revealed no statistically significant differences between groups, thereby confirming their comparability at baseline and reinforcing the internal validity of the group comparisons presented.

### 3.2. Functional Tests

The results of the linear mixed model analysis of 30s-CS test, presented in Table 2, shows the significant effect of the factor TIME (F(1,21) = 9.215, *p* = 0.006, ƞ^2^ = 0.305) indicating a significant increase from PRE to POST. No significant main effect of GROUP and no significant TIME × GROUP interactions were found. In addition, TUG time duration was significantly lower in POST than PRE (TIME: F(1,21) = 5.786, *p* = 0.025, ƞ^2^ = 0.216), while no other significant effects were found.

The statistical analysis on CSR tests performed with the right leg revealed a significant increase effect of GROUP (F(2,21) = 3.848, *p* = 0.038, ƞ^2^ = 0.268) indicating that SS reported a higher improvement with respect other groups and no significant effects of TIME were found. Post hoc tests showed that SS was significantly lower in PRE with respects to CG (*p* = 0.013) and DS (*p* = 0.009), indeed SS demonstrated a significant increase between PRE and POST (*p* = 0.007) (TIME × GROUP: F(2,21) = 3.307, *p* = 0.043; ƞ^2^ = 0.240).

The CSR performed with the left leg reported no significant effect in TIME; whereas a significant effect of GROUP was found (F(2,21) = 7.217, *p* = 0.004; ƞ^2^ = 0.407) indicating that SS reported a higher improvement respect CG and DS. Furthermore, a significant TIME × GROUP appeared (F(2,21) = 6.816, *p* = 0.005; ƞ^2^ = 0.394). The results of post hoc tests showed significant differences in PRE between SS and both CG (*p* = 0.0001) and DS (*p* = 0.001). In addition, the results indicated that left leg’s CSR performance significantly improved only in SS (*p* = 0.001).

### 3.3. Ankle Motion Analysis

The results of the linear mixed model analysis of ankle parameters during stair descent are presented in Table 2. The ankle angle during the initial foot contact revealed no significant interaction in TIME (F(1,21) = 3.763, *p* = 0.066, ƞ^2^ = 0.043), GROUP (F(2,21) = 0.643, *p* = 0.536, ƞ^2^ = 0.192), and TIME × GROUP (F(2,21) = 0.146, *p* = 0.865, ƞ^2^ = 0.175).

The ankle angle in single support was significantly higher in POST than in PRE (TIME: (F(1,21) = 5.008, *p* = 0.036; ƞ^2^ = 0.152), while no effects of GROUP (F(2,21) = 1.156, *p* = 0.334, ƞ^2^ = 0.058) and TIME × GROUP (F(2,21) = 0.083, *p* = 0.92, ƞ^2^ = 0.014) were observed.

The angle in the final foot contact position showed a significant effect of TIME × GROUP (F(2,21) = 4.546, *p* = 0.023; ƞ^2^ = 0.305), reporting a significant difference between PRE and POST only in the SS (*p* = 0.002). No significant effect of TIME (F(1,21) = 4.284, *p* = 0.051, ƞ^2^ = 0.045) and GROUP (F(2,21) = 1.272, *p* = 0.301, ƞ^2^ = 0.105) were found.

The statistical analysis of single support duration indicated no significant effects of TIME (F(1,21) = 0.950, *p* = 0.341, ƞ^2^ = 0.178), GROUP (F(2,21) = 2.495, *p* = 0.107, ƞ^2^ = 0.105), and TIME × GROUP (F(2,21) = 2.223, *p* = 0.133, ƞ^2^ = 0.305).

### 3.4. Rating of Perceived Exertion

The statistical analysis on RPE*post warm-up* (Table 3) did not report significant effects (TIME: F(3,179) = 0.098 *p* = 0.961; GROUP: (F(2,84) = 0.601, *p* = 0.550; TIME × GROUP: (F(6,84) = 0.143, *p* = 0.990). RPE*post exercise* showed a significant effect of TIME (F(3,84) = 47.342, *p* = 0.0001, ƞ^2^ = 0.33) and GROUP (F(2,24) = 10.967, *p* = 0.0001, ƞ^2^ = 0.64). Furthermore, a significant interaction of TIME × GROUP was found (F(6,63) = 4.203, *p* = 0.001, ƞ^2^ = 0.27). For CG, RPE RPE*post exercise* in the first week was significantly lower than both the third (*p* = 0.001) and fourth (*p* = 0.016); RPE*post exercise* values in the second week were significantly lower than the third and fourth weeks (*p* = 0.0001). The DS group reported that RPE*post exercise* in both the first and second weeks were significantly lower than the third (*p* = 0.0001 either) and fourth weeks (*p* = 0.006 and *p* = 0.0001). In addition, the SS group reported lower values in the first and second weeks than the third (*p* = 0.0001, always) and fourth weeks (*p* = 0.002 and *p* = 0.0001, respectively). In the first week, RPE-*post exercise* values of both DS and SS groups were significantly lower than the CG (*p* = 0.0001 and *p* = 0.001, respectively). In the second week RPE*post exercise* of the CG were significantly higher compared with DS (*p* = 0.0001) and SS (*p* = 0.003). In the third week, the CG were significantly higher compared to the DS (*p* = 0.0001) and SS (*p* = 0.007). Finally, at the fourth week, both DS and SS reported significantly lower values than CG (*p* = 0.0001 and *p* = 0.002, respectively).

## 4. Discussion

This study evaluated whether combining stretching exercises and progressive RT with elastic bands could be effective in improving functional capacity and ankle joint control during stair descent in active older women. To this end, we compared three training protocols differentiated by the three warm-up routines: SS exercises (SS group), DS exercises (DS group) and walking (CG group).

Our findings showed that, in active older women, only SS prior to the progressive RT improved CSR performance in both legs as well as the ankle angle in final foot contact position, while RT alone improved lower limb strength and functional mobility across all groups. Moreover, both stretching modalities were effective in reducing perceived physical fatigue, particularly during the initial training phase, and enhancing exercise tolerance during training sessions. However, it should be noted that these results cannot be generalized to the entire elderly population, as there is a lack of a male group.

### 4.1. Functional Performance and Strength Outcomes

Our study confirms the well-documented effectiveness of progressive RT with elastic bands in enhancing functional capacity in active older adults. Elastic bands provide strength and joint function improvements compared to traditional RT methods, especially in peak torque in dorsiflexion and plantarflexion [18]. Moreover, elastic band training is particularly attractive to older women due to its accessibility, safety, and suitability for use in non-equipped environments [15,18,21,22].

All groups, including the CG, which performed only RT without stretching within the warm-up, showed significant improvements in the 30s-CS test and the TUG test. These improvements, observed across all groups, underscore the value of elastic band training as a viable and accessible form of resistance exercise for aging populations [15,16,22]. This supports evidence that even short-term, low-load RT can induce significant neuromuscular adaptations, thus leading to improvements in lower limb strength, mobility, and balance stability in older adults. These findings are consistent with those reported in a previous study [47] conducted in older men, suggesting that similar neuromuscular benefits can be achieved regardless of sex under comparable training conditions.

However, the absence of significant interaction effects between groups for the 30s-CS and TUG tests suggests that adding stretching (whether static or dynamic) to the warm-up did not produce additional measurable benefits in these aspects of functional performance.

The CSR test revealed significant gains in the SS group compared to the DS and CG groups. In particular, the SS group showed improvements of approximately eight cm in both legs compared to both DS and CG.

This significant improvement in CSR performance reinforces the notion that static stretching is particularly effective in enhancing range of motion (ROM) and flexibility, particularly within the posterior kinetic chain [23,48,49]. This gain, approximately eight cm for both lower limbs, indicates that SS, with its emphasis on sustained muscle elongation, may produce greater improvements in muscle-tendon unit flexibility than dynamic stretching [50]. This improvement in CSR can be attributed to mechanical adaptations, such as reduced stiffness in musculotendinous and muscular joint structures, and neural factors like increased stretch tolerance and a reduced motor neurons excitability [27]. Given the strong relationship between joint flexibility and postural control during a dynamic tasks such as stair descent [4,8], these findings highlight the effectiveness of SS in the warm-up for older populations. The decline in muscle strength, walking speed and flexibility is a major contributor to mobility limitations and an increased risk of falls in the older population [51], which negatively impacts the maintenance of independence and quality of life [8,18]. In this context, the role of SS, particularly for the muscles of the posterior chain (i.e., the hamstrings), should not be underestimated. These muscles are crucial for safe and efficient walking, as well as for dealing with everyday obstacles such as stairs.

Overall, our findings indicate that SS may be more effective than DS in older adults. This could be explained as SS better addressing age-related muscle stiffness and reduced stretch tolerance, which are less responsive to DS. These age-related changes in the musculotendinous system make sustained elongation techniques more suitable for improving flexibility and joint control in aging populations.

### 4.2. Ankle Motion Analysis

Stair descent poses high biomechanical demands due to eccentric control requirements [9]. The current high risk of falling can be attributed to limited ankle motion, which predisposes to a ‘controlled fall’, and limited ankle strength development, which includes the individual’s ability to respond to unexpected perturbations when stepping down. In our study, progressive RT contributed to differences in balance control by strengthening the ankle and hip muscles. In addition, stretching exercises relaxed and lengthened the muscles involved in resistance training, improving stiffness and developing higher joint moments [8,28].

Our motion analysis of the dorsiflexion ankle during the stair descent provided new insights into the biomechanical effects of the stretching interventions. Although changes in initial foot contact and single support phases did not reach statistical significance, the final foot contact angle improved significantly only in the SS group. This change reflects enhanced dorsiflexion control during the eccentric phase of stair descent, and it is particularly relevant for fall prevention in older adults, as better dorsiflexion contributes to safer and more stable step negotiation. This suggests that enhanced dorsiflexion capacity, which influences ankle range of motion [52], may facilitate better control of the ankle joint during the critical eccentric phase of stair descent, in which ankle musculature plays a key role in absorbing and dissipating mechanical loads, which is essential for the controlled lowering of the limb and shock absorption during stair descent [25]. These kinematic improvements likely reflect both increased flexibility and improved eccentric control in the ankle joint, a result supported by prior evidence that stretching and RT synergistically improve joint control and reduce fall risk during locomotor tasks [7,25]. This aspect is particularly important in older adults, as improved dorsiflexion can lead to better shock absorption and stability during weight-bearing activities, thus reducing the risk of falls [8,53]. Interestingly, the DS group did not exhibit comparable improvements in either ROM or motion analysis parameters, despite previous reports suggesting dynamic stretching can enhance acute functional performance [52]. One explanation may be that the older participants in this study, already physically active, may have derived minimal incremental benefit from DS, which is generally more effective for enhancing performance in younger or athletic populations [37]. Given that stair descent is a high-risk activity for older adults, often leading to falls due to compromised eccentric strength and flexibility, this result has meaningful implications. Improved ankle dorsiflexion range and control likely reduce the mechanical demand on the joint and enhance stability, thus contributing to fall prevention. The lack of significant interaction effects between groups further suggests that while both SS and DS improve functional capacity, static stretching may be more beneficial for enhancing specific aspects of joint mobility, particularly in tasks requiring controlled, sustained movements like stair descent.

### 4.3. Perceived Exertion and Adherence

RPE results highlighted an interesting aspect of the intervention. The SS group reported higher perceived exertion during the initial weeks, which could be due to the unfamiliarity with static stretching exercises and the discomfort associated with reaching the POD. However, the significant decrease in RPE over time in the SS group suggests that participants adapted to the intensity of the exercises, likely due to increased flexibility and reduced muscle stiffness [40,54]. The DS group consistently reported lower RPE scores, indicating that dynamic stretching might be perceived as less strenuous, potentially improving adherence to such protocols in long-term exercise programs [55].

However, despite DS being perceived as less strenuous, it did not yield significant improvements in flexibility or ankle kinematics; thus, suggesting a trade-off between comfort and efficacy and reinforcing the idea that lower exertion is not always related to improvements in functional outcomes.

These findings are consistent with previous work highlighting the importance of perceived exertion in supporting long-term adherence to home-based resistance training programs in older adults [56] and further suggest that RPE is also a useful and effective tool for monitoring exercise intensity in active older women.

Finally, the lower RPE reported by both the SS and DS groups during the first two weeks may reflect early neuromuscular adaptation and increased psychological readiness, marking the initial phases of motor learning.

#### 4.3.1. Limitations and Future Directions

This study provides valuable insight into the effects of stretching on functional capacity and ankle biomechanics, however, there are several limitations to consider. Although the sample size was sufficient to detect significant differences, the sample was relatively small and only included older women. This restricts the generalizability of our findings, particularly regarding potential gender differences in response to the interventions. Future studies should include both men and women to enable direct comparisons between the sexes, and to improve our understanding of how these interventions affect older adults of different genders and levels of physical fitness.

In addition, the study focused on a short-term intervention period of four weeks. Long-term studies are needed to determine whether the observed benefits are sustained over time and how they impact the overall mobility and quality of life of older adults. In addition, the incorporation of more advanced movement analysis tools, such as three-dimensional gait analysis, may provide a more complete understanding of the biomechanical changes induced by stretching interventions.

#### 4.3.2. Practical Applications

The results of this study demonstrated significant improvements in lower-limb flexibility, mobility, and stair descent efficiency in active older women. Therefore, it is crucial to emphasize the importance of incorporating SS exercises into the warm-up routine before progressive lower-limb elastic band training in adapted physical activity sessions for active older women. This training led to significant improvements in lower-limb flexibility and ankle joint control during stair descent, which are key components in fall prevention. These findings support the integration of SS into pre-activity routines to promote joint mobility and improve the safety and efficacy of stair navigation in active older women.

## 5. Conclusions

In conclusion, static stretching (SS) prior to progressive RT resulted in greater gains in lower-limb flexibility and improved ankle kinematics during stair descent in active older women. These findings highlighted the value of static stretching as a warm-up strategy for increasing joint range of motion and potentially reducing the risk of falls in this specific population. Moreover, both stretching modalities were effective in reducing perceived physical fatigue and enhancing exercise tolerance during training sessions. However, participants who performed dynamic stretching (DS) reported lower RPE values, suggesting that this approach may be more suitable for older individuals beginning resistance training with elastic bands.

Together, these results support the idea of a tailored approach to warm-up routines, based on individual goals, whether that is prioritizing compliance after training, or optimizing mobility and functional movement.

## Figures and Tables

**Table 1:** Subjects’ anthropometric characteristics at baseline. Data are means ± standard error.

	DS (n = 8)	SS (n = 8)	CG (n = 8)	*p*-Values
***Height (m)***	159.62 ± 2.88	157.37 ± 2.32	160.75 ± 2.25	n.s.
***Body Mass (kg)***	65.67 ± 7.39	63.11 ± 3.61	66.32 ± 2.93	n.s.
***BMI (kg/m^2^)***	25.88 ± 2.19	26.56 ± 1.37	27.38 ± 1.22	n.s.
***Skeletal muscle mass (kg)***	40.48 ± 2.45	39.33 ± 1.24	41.51 ± 1.05	n.s.
***HGS (kg)***	17.98 ± 0.80	17.96 ± 0.75	17.98 ± 0.78	n.s.

BMI: body mass index; HGS: handgrip strength; n.s.: not significant.

**Table 2:** Functional tests and ankle motion analysis parameters of DS, SS, and CG groups before and after the intervention. Data are means ± standard error.

	DS		SS		CG	
	PRE	POST	PRE	POST	PRE	POST
***Functional tests***						
*30s-CS (reps)*	11.88 ± 1.35 (10.94, 12.80)	13.00 ± 1.19 (12.06, 13.93)	12.00 ± 0.53 (11.06, 12.93)	13.25 ± 1.98 (12.32, 14.18)	11.62 ± 1.19 (10.95, 12.55)	11.88 ± 1.12 (10.94, 12.80)
*TUG (s)*	7.93 ± 0.35 (7.59, 8.28)	7.75 ± 0.27 (7.41, 8.09)	8.03 ± 0.48 (7.69, 8.38)	7.88 ± 0.45 (7.54, 8.22)	8.01 ± 0.62 (6.67, 8.36)	8.02 ±0.57 (7.69, 8.38)
*CSR right leg (cm)*	2.25 ±6.36 (−2.43, 6.93)	0.25 ± 0.71 (−4.43, 4.93)	−8.12 ± 8.93 (−12.8, −3.45)	−0.75 ± 1.49 (−5.43, 3.93)	1.79 ± 6.94 (−2.89, 6.46)	4.00 ± 9.14 (−0.68, 8.68)
*CSR left leg (cm)*	1.88 ±5.30 (−2.81, 6.56)	0.00 ± 0.11 (−4.68, 4.67)	−10.88 ± 10.75 (−15.5, −6.19)	−2.25 ± 6.36 (−6.93, 2.43)	4.25 ± 4.62 (−0.43, 8.93)	3.75 ± 6.94 (−0.93, 8.43)
***Ankle motion analysis***						
*Initial foot contact (°)*	133.68 ± 4.51 (128.64, 138.71]	135.14 ± 4.84 (130.10, 140.17)	136.58 ± 5.95 (131.64, 141.60)	138.06 ± 6.33 (133.02, 143.09)	132.6 ± 10.22 (127.57, 137.63)	135.16 ± 8.02 (130.12, 140.19)
*Single support (°)*	107.66 ± 5.68 (102.38, 112.94)	109.96 ± 5.60 (104.68, 115.24)	111.9 ± 8.75 (106.62, 117.18)	115.54 ± 10.34 (110.25, 120.82)	110.42 ± 6.59 (105.14, 115.70)	113.52 ± 5.59 (108.24, 118.8)
*Final foot contact (°)*	83.80 ± 8.90 (77.37, 90.23)	83.31 ± 7.19 (76.88, 89.74)	85.80 ± 7.44 (79.37, 92.23)	94.41 ± 11.78 (87.98, 100.84)	87.00 ± 9.72 (80.57, 93.43)	87.30 ± 7.28 (80.91, 93.77)
*Single support duration (s)*	1.72 ± 0.44 (1.45, 1.98)	1.58 ± 0.20 (1.31, 1.84)	1.92 ± 0.24 (1.66, 2.19)	2.04 ± 0.52 (1.77, 2.31)	1.78 ± 0.42 (1.52, 2.05)	2.04 ± 0.28 (1.77, 2.30)

DS: dynamic stretching group; SS: static stretching group; CG: control group; 30s-CS: 30 s Chair Stand test; TUG: Time Up and Go test; CSR: Chair Sit and Reach.

**Table 3:** The 4-week RPE values after warm-up and exercise. Data are median ± standard error.

	DS	SS	CG
***RPE at 1st week***			
POST_warm-up_	2.62 ± 0.52 (2.36, 2.88)	2.56 ± 0.32 (2.30, 2.82)	2.62 ± 0.23 (2.36, 2.88)
POST_exercise_	4.62 ± 0.52 (3.99, 5.25)	4.81 ± 0.26 (4.19, 5.44)	6.50 ± 0.53 (5.87, 7.12)
***RPE at 2nd week***			
POST_warm-up_	2.50 ± 0.53 (2.24, 2.76)	2.56 ± 0.32 (2.30, 2.82)	2.68 ± 0.26 (2.42, 2.95)
POST_exercise_	4.38 ± 0.44 (3.75, 5.00)	4.62 ± 0.35 (4.00, 5.25)	6.12 ± 0.35 (5.50, 6.75)
***RPE at 3rd week***			
POST_warm-up_	2.56 ± 0.42 (2.30, 2.82)	2.50 ± 0.29 (2.24, 2.76)	2.56 ± 0.32 (2.3, 2.82)
POST_exercise_	5.50 ± 0.53 (4.87, 6.13)	5.94 ± 0.32 (5.31, 6.56)	7.31 ± 0.26 (6.69, 7.94)
***RPE at 4th week***			
POST_warm-up_	2.56 ± 0.42 (2.30, 2.82)	2.50 ± 0.25 (2.24, 2.76)	2.62 ± 0.23 (2.36, 2.88)
POST_exercise_	5.31 ± 0.37 (4.69, 5.94)	5.56 ± 0.49 (4.94, 6.19)	7.12 ± 0.23 (6.49, 7.75)

DS: dynamic stretching group; SS: static stretching group; CG: control group; RPE: rating of perceived exertion.

## Data Availability

Data generated or analyzed during this study are available from the corresponding author upon reasonable request.
